# A novel approach to establish orthotopic right lung transplantation in rats

**DOI:** 10.1016/j.jhlto.2025.100407

**Published:** 2025-10-10

**Authors:** Ye Wu, Xinyue Zhang, Yang Li, Yang Zhao, Jing Chen, Xiaohan Jin, Jier Ma, Xiangyun Zheng, Dan Chen, Masaaki Sato, Hongtao Tang, Dong Tian

**Affiliations:** a Department of Thoracic Surgery, West China Hospital, Sichuan University, Chengdu, China; b Department of Cardiothoracic Surgery, The First Affiliated Hospital of Chongqing Medical University, Chongqing, China; c Lung Transplant Research Laboratory, Institute of Thoracic Oncology, West China Hospital, Sichuan University, Chengdu, China; d School of Clinical Medicine, West China School of Medicine, Sichuan University, Chengdu, China; e School of Clinical Medicine, North Sichuan Medical College, Nanchong, China; f Department of Thoracic Surgery, The University of Tokyo Hospital, Tokyo, Japan; g Department of Cardiovascular Surgery, West China Hospital, Sichuan University, Chengdu, China

**Keywords:** right lung transplantation, animal model, rat, cuff technique, Pendulum model

## Abstract

**Background:**

Although the left lung transplantation model in rats is widely adopted, the right lung transplantation remains less common due to anatomical complexity and technical challenges. As the right lung contributes approximately 70% of total lung function, the orthotopic right lung transplantation model provides a more physiological and clinically relevant platform for evaluating graft function, particularly during the survival period, compared to the commonly used left lung model. In this study, we developed a novel orthotopic right lung transplantation technique based on our previous “pendulum model” in rats, incorporating several innovations to enhance accessibility for beginners and provide a more feasible and reliable platform for basic research.

**Methods:**

Twenty consecutive orthotopic right lung transplantation were performed on Sprague-Dawley rats by a single surgeon using our modified techniques. The duration of each procedure, ischemia time, post-surgery complications, survival rate at 2 weeks, and histological staining for graft evaluation were recorded.

**Results:**

The key innovations of our novel model mainly include extending the donor’s right pulmonary vein by ligating the left branch, improving the visibility by integrating anterior and posterior surgical fields of the right pulmonary hilum, and modifications to the incision of vessels. All transplantations were successfully completed without bronchial or vascular tearing, twisting, or folding. The heart-lung block retrieval, donor lung preparation, cold ischemia time, anastomosis time, and total operation times were 7.5 ± 0.3 minutes, 22.6 ± 1.8 minutes, 38.3 ± 1.6 minutes, 20.2 ± 1.3 minutes, and 76.1 ± 2.2 minutes, respectively. During the follow-up, only 2 rats (10%) succumbed to complications related to atelectasis and infection. Histological findings showed preserved pulmonary architecture without evidence of acute injury, infection, or other pathological abnormalities.

**Conclusion:**

Our novel technique streamlined the learning process for rat right lung transplantation and yielded reliable outcomes, suggesting a feasible option for basic research in lung transplantation.

## Background

Lung transplantation (LTx) has been known as the only effective treatment for patients with end-stage pulmonary diseases.^1^, ^2^ As the International Society for Heart and Lung Transplantation (ISHLT) reported in 2021, more than 5,000 LTx procedures have been performed worldwide, and an increasing trend has been maintained annually.^3^ The development of various animal models has contributed to investigations into the complications associated with lung transplantation, such as ischemia/reperfusion injury and chronic lung allograft dysfunction.^4^, ^5^, ^6^, ^7^, ^8^, ^9^, ^10^ Among these models, rats are superior in terms of the balance between cost and physical similarities to humans.^11^

The orthotopic rat left LTx model is widely adopted by researchers. Since Mizuta first introduced the cuff technique for rat left LTx,^12^ numerous studies have refined and improved the model. Notable advancements include our previous “pendulum model” and the recent modification by Li et al., which utilized waist-shaped cuffs to prevent slippage.^13^, ^14^ These improvements have significantly increased the model’s success rate and reproducibility.^15^

However, researchers have observed that recipient animals can survive and appear healthy even with left lung allograft dysfunction.^16^ As the right lung compensates for approximately 70% of the total lung function,^17^ the orthotopic right lung transplantation (RLTx) model is more representable to reflect graft function throughout the survival period.^11^ Kawaguchi et al. pioneered the successful establishment of a rat model of right lung transplantation, demonstrating that recipients could survive solely on the allograft following left pneumonectomy.^18^ Subsequent studies compared this approach with left lung transplantation followed by right pneumonectomy, revealing significantly higher survival rates in the RLTx group. This finding underscores the right lung’s greater volume and functional capacity, making it a more accurate indicator of graft function in rat models. However, the procedure is technically demanding due to the complex anatomy between the right pulmonary vein (PV) and pulmonary artery (PA) branches, as well as the necessity for separate anastomosis of 2 PV branches, resulting in extended operative time and ischemic periods of up to 69 ± 8 minutes.^18^ Li et al. later modified the model in mice by ligating the upper branch and anastomosing only the lower branch, achieving a 7.5% increase in success rates.^19^ To simplify the procedure, we previously developed a RLTx model using a left-to-right inverted anastomosis technique, which effectively shortened ischemic time, improved reproducibility, and provided a feasible platform for studies related to inverted lung transplantation.^20^ Despite these advancements, the operative duration and success rate of orthotopic RLTx remain suboptimal, with even experienced surgeons facing challenges such as transplant failure due to extended ischemic times. The RLTx model is yet to be optimized.

This study introduces a modified orthotopic rat RLTx model based on our "pendulum model" and the concept of "left-to-right inverted RLTx". By integrating refined surgical techniques, we aim to establish a stable model characterized by simple procedures, reduced ischemic time, and improved success rates.

## Material and methods

### Animals and ethical approval

Forty male Sprague-Dawley (SD) rats (7–8 weeks old, weighing around 300 g, SiPeiFu, Beijing, China) were housed at room temperature (22 ± 1℃) with a 12/12 hours light/dark cycle and free access to food and water ad libitum 7 days prior to the operation. The rats were returned to the same environment post-transplantation. All the surgeries were performed at the Lung Transplant Research Laboratory, Institute of Thoracic Oncology, West China Hospital, Sichuan University. This study was approved by the Experimental Animal Ethics Committee of the West China School of Medicine, Sichuan University (NO. 20221229003).

### Donor procedure

The methods for tracheal intubation and en bloc retrieval of the heart-lung block were adapted from established protocols in prior studies.^16^, ^19^ In brief, following induction of anesthesia (isoflurane 3–5% for induction, RWD Life Science, Shenzhen, China),^21^ intubation was performed, and the donor rat was mechanically ventilated with room air during the procedure. The chest, abdominal, and anterior neck skin were partially removed. A median laparotomy was performed up to the xiphoid process, which was subsequently transected to improve exposure. The diaphragm was then incised along the costal margin to expose the inferior vena cava. Systemic heparinization was achieved by administering heparin sodium (Cat# S1346, Selleckchem) intravenously through the inferior vena cava at a dose of 1,000 IU/kg. The ribs adjacent to the sternum were transected bilaterally, and a portion of the thymus tissue was excised. The ribs were then everted to maximize exposure of the thoracic cavity. The abdominal vein and inferior vena cava were sequentially transected. The left atrial appendage and the PA root were rapidly incised, and the perfusion solution (Celsior, Institut Georges Lopez, France) was slowly and steadily injected into the PA incision until the donor lungs turned predominantly white. Upon reaching 50% of their maximal ventilatory volume, the trachea was clamped, and the heart-lung block was retrieved (Video 1).

Supplementary material related to this article can be found online at doi:10.1016/j.jhlto.2025.100407.

The following is the Supplementary material related to this article Video 1..

Video 1

### Preparation of the donor lung

Cuffs were fabricated using 14 G venous catheters (a diameter of 2 mm for the pulmonary artery and vein and 2.1 mm for the bronchus), with body and tail lengths of 1 mm for the pulmonary vein and 1.5 mm for the bronchus and artery. Donor lung preparation was performed under hypothermic conditions. The right main bronchus was transected at the tracheal carina, and the right PA was identified via the left PA and subsequently transected.

**Figure 1:**
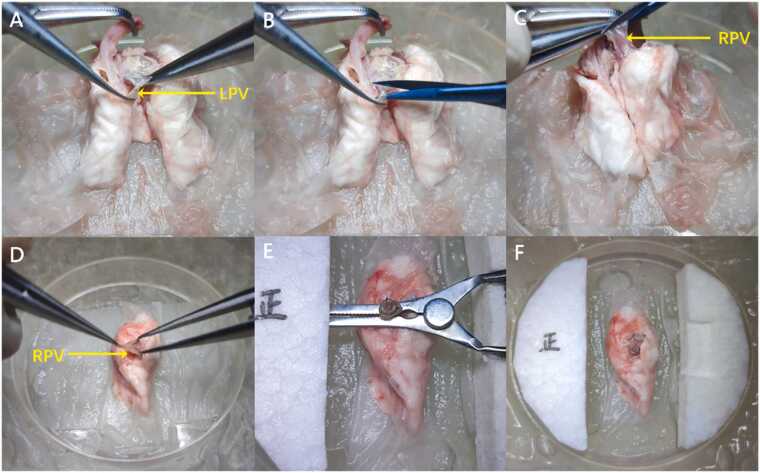
Preparation of donor lung for vascular elongation. (A) The LPV was ligated at its proximal (cardiac) end. (B) The LPV was transected distally at the ligation point. (C) The RPV was divided together with a portion of the left atrial wall. (D) The RPV, bronchus, and right pulmonary artery were sequentially dissected (shown here for the RPV). (E) The RPV, bronchus, and right pulmonary artery were sequentially prepared with cuffs (shown here for the RPV). (F) Following cuff preparation, the donor lung was preserved under 4°C hypothermic conditions. LPV, left pulmonary vein; RPV, right pulmonary vein.

To facilitate anastomosis, a novel surgical technique was introduced to elongate the PV of the donor lung. The left PV was dissected, ligated at its proximal end, and transected distally at the ligation point, eliminating length restrictions imposed by the left PV (Figure 1A, B). The right PV was then transected along with a portion of the left atrium, allowing the 2 branches of the right PV to merge (Video 2, Figure 1C). The hilar of the donor lung was meticulously dissected, and cuffs for the PV, right main bronchus, and PA were prepared following established methodologies.^13^, ^20^ The cuff tail of the vascular was oriented toward the anterior margin of the donor lung, characterized by a thinner and sharper border. The bronchial cuff tail was directed toward the posterior margin, which appears thicker and more rounded (Video 3, Figure 1D, E). Finally, the donor lung was stored in a 4°C hypothermic environment for subsequent use (Figure 1F).

Supplementary material related to this article can be found online at doi:10.1016/j.jhlto.2025.100407.

The following is the Supplementary material related to this article Video 2 and Video 3..

Video 2

Video 3

### Recipient procedure

Following induction of anesthesia in the recipient rat, orotracheal intubation was performed using a custom-designed intubation device, with anesthesia maintained at a concentration of 5 ppm. Ventilation parameters were set to a respiratory rate of 90 breaths/min and a tidal volume of 2.5–3 ml (approximately 1 ml per 100 g). Upon opening the thoracic cavity at the cardiac apex, the anesthetic concentration was reduced to 3 ppm. A translational eyelid opener was utilized to widen the incision, and sterile cotton swabs were employed to retract the lobes of the right lung (cranial, middle, caudal, and accessory).^22^ The cranial and accessory lobes were ligated and excised, and the right hilar structures were stabilized using a nondestructive sidewall forceps. From left to right, the vein (including its inferior and superior branches) and artery were identified, with the bronchus located posterior to the vascular plane. The tidal volume was appropriately reduced to prevent hyperinflation of the contralateral lung. From an anterior view, the hilar was meticulously dissected to enlarge the space between the superior branch of the PV and the PA. The superior branch of the PV was ligated and transected. A Stevens nerve hook was used to dissect the overlapping region among the PV, PA, and bronchus. Finally, the bronchus and adjacent tissue near the proximal end were dissected from a posterior view.

**Figure 2:**
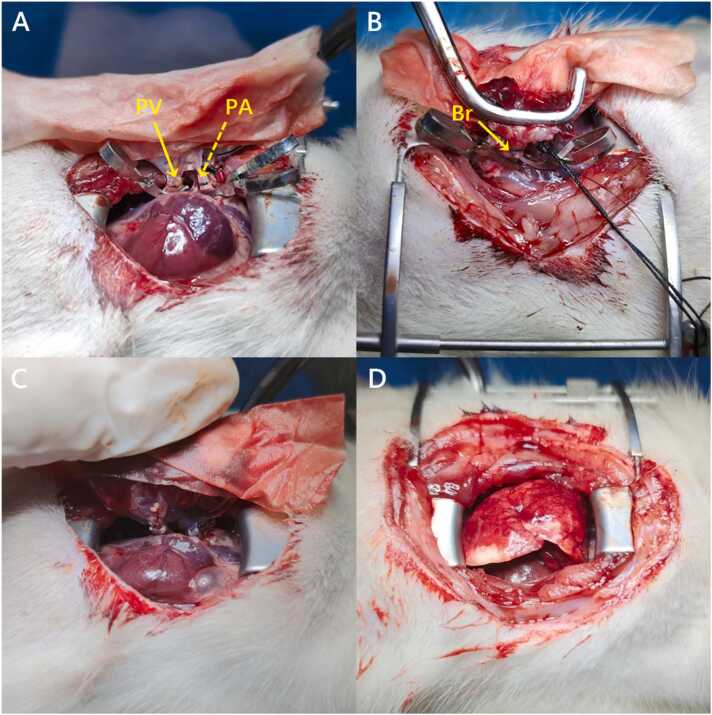
Right lung transplantation procedure in rats. (A) Anterior view: Completed anastomoses of the inferior branch of the PV (solid arrow) and PA (dashed arrow). (B) Posterior view: Completed Br anastomosis with surrounding tissue approximation (solid arrow). (C) Reperfusion phase: Restoration of blood flow and ventilation through the transplanted lung (3–5 minutes duration). (D) Final positioning: The transplanted lung properly situated within the recipient's thoracic cavity. Br, bronchial; PA, pulmonary artery; PV, pulmonary vein.

During the anastomosis, we introduced an innovative surgical approach involving sequential anastomosis of the PV and PA from the anterior aspect, followed by bronchial anastomosis from the posterior aspect. After achieving sufficient anatomical exposure of the right hilar, 2 microvascular clamps were applied to the proximal segments of the PA and PV from an anterior view. A transverse incision was made on the anterior wall of vessels distally to allow drainage of blood. The donor lung was retrieved from the hypothermic condition and positioned using another nondestructive sidewall forceps. An 8–0 Prolene knot was placed loosely around the vascular. The PV cuff is clamped at the distal end, then inserted as deeply as possible into the venous lumen, close to the proximal end of the heart. Due to the relatively lengthened distance between the cuff and the vascular incision in RLTx, careful adjustment of the donor lung position is required to avoid dislodge. Then, the PV and the cuff body were secured by tightening of the knot. The PA was anastomosed in a similar manner (Figure 2A). The graft was then tilted forward and switched to the reverse view plane for bronchus anastomosis (Figure 2B). In cases of a large bronchial lumen, after the cuff is inserted, microvascular clamps were used to secure the cuff tail to the bronchus, preventing slippage prior to ligation (Video 4). Finally, the microvascular clamps were removed to restore ventilation and blood flow, followed by 3–5 minutes of reperfusion. The chest was closed and the skin was sutured once the right lung was fully expanded and hemodynamic stability was confirmed (Video 5, Figure 2C, D).

Supplementary material related to this article can be found online at doi:10.1016/j.jhlto.2025.100407.

The following is the Supplementary material related to this article Video 4 and Video 5..

Video 4

Video 5

### Postoperative care and assessment

Following transplantation, meloxicam was administered subcutaneously at 1 mg/kg.^21^ Throughout the follow-up period, the recipient rats were maintained on a standard diet, with regular monitoring of activity levels, respiratory status, and feeding behavior.

All recipient rats were sacrificed 2 weeks after transplantation. After sacrifice, the transplanted right lung grafts and the native right lung tissue from non-transplanted controls were collected, fixed, embedded, sectioned, and subjected to hematoxylin–eosin (HE) staining. Random HE staining fields were selected (objective magnification, 4× or 10×).

## Results

### Key modifications and techniques of the model

**Figure 3:**
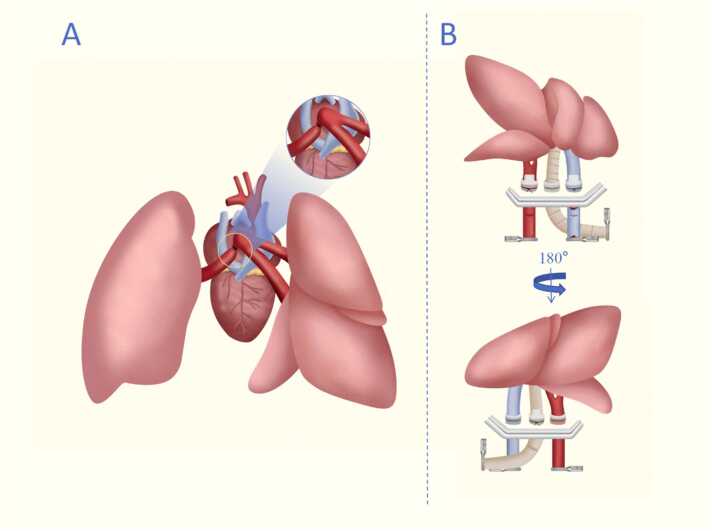
Schematic drawing of modified orthotopic right lung transplantation technique. (A) During donor preparation, the left pulmonary vein is dissected, ligated at its proximal end, and then transected distally at the point of ligation. (B) Recipient Procedure includes sequentially anastomosing the pulmonary vein and pulmonary artery from the anterior aspect, followed by anastomosis of the bronchus from the posterior aspect. Thereafter, a transverse incision was made at the distal end to allow for the drainage of blood.

To optimize the surgical outcome and ensure model stability, several key technical refinements were implemented:

(1) The left PV was ligated and dissected proximally to the convergence site. Subsequently, the right PV was transected along with a portion of the left atrium. This facilitates the convergence of right PV branches and extends the available length for cuff preparation (Figure 3A, Figure S1).

(2) To minimize the risk of vascular tearing, anterior-view was adopted for the anastomosis of both the PV and PA, improving visibility and optimizing surgical precision. Posterior-view anastomosis was applied for the anastomosis of the bronchial to help maintain anatomical orientation and prevent torsion and misalignment (Figure 3B).

(3) Precise transverse incisions were made in the PV, PA, and right main bronchus, which improved the overall procedural stability and significantly reduced the risk of vessel tearing during implantation.

### Surgical outcomes

A total of 20 (100%) consecutive orthotopic RLTx were successfully performed, with no instances of tearing, distortion, or folding in the bronchi or blood vessels. During the 2-week follow-up period, 2 cases died, with one case died of pulmonary atelectasis 7 days after surgery and another case died of infection 10 days after surgery, as confirmed by autopsy. The remaining 18 rats (90%) exhibited no complications or mortality.

### Operation time of each procedure

The duration of each procedure, including heart-lung block retrieval, donor lung preparation, cold ischemia time, anastomosis time, and total surgical time, were 7.5 ± 0.3, 22.6 ± 1.8, 38.3 ± 1.6, 20.2 ± 1.3, and 76.1 ± 2.2 minutes, respectively (Table 1).

**Table 1:** Operation Time of Each Transplantation Procedure

Transplantation procedure^a^^,^^b^^,^^c^^,^^d^^,^^e^	Operation time (minutes)
Heart-lung block retrieval	7.5 ± 0.3
Donor lung preparation	22.6 ± 1.8
Cold ischemia	38.3 ± 1.6
Anastomosis time	20.2 ± 1.3
Total surgical time	76.1 ± 2.2

a Heart-lung block retrieval: Time interval from donor midline sternolaparotomy to en bloc heart-lung harvest.

b Donor lung preparation: Time interval from en bloc heart-lung harvest to completion of donor lung cuff preparation.

c Cold ischemia time: Time interval from flushing the donor lungs in situ to graft removal from hypothermia storage.

d Anastomosis time: Time interval from removal of donor lungs from cold storage to reperfusion.

e Total surgical time: Time interval from initiation of donor midline sternolaparotomy to completion of recipient incision closure.

### Histology analysis

**Figure 4:**
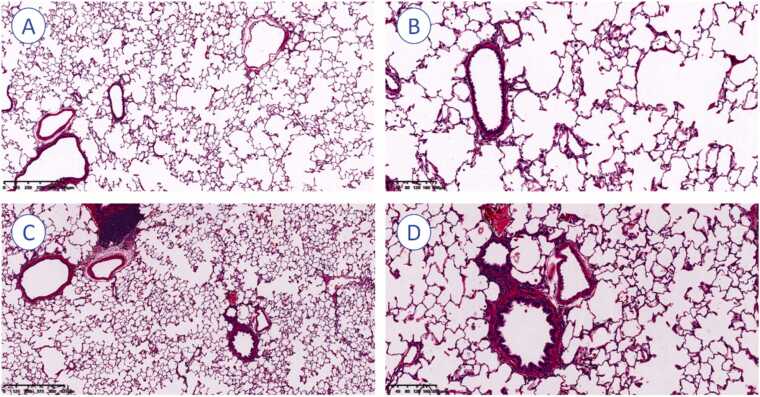
Histological assessment of lung tissue with hematoxylin–eosin (HE) staining. (A) Right lung graft at 14 days post-transplantation (×4). (B) Right lung graft at 14 days post-transplantation (×10). (C) Native right lung tissue from non-transplanted controls (×4). (D) Native right lung tissue from non-transplanted controls (×10). Pulmonary parenchyma, architecture, and alveolar structures were preserved, with no evidence of rejection observed in any rats after syngeneic transplantation.

Histological analysis demonstrated preserved alveolar architecture and intact bronchial epithelium without evidence of detachment or edema. Vascular walls appeared normal, without edema or thickening, and alveolar spaces were largely free of exudates and erythrocyte aggregation. Mild emphysematous changes were noted in the transplanted lungs. No inflammatory cell infiltration, necrosis, or fibrotic remodeling was observed, and the pulmonary parenchyma otherwise remained intact without signs of acute injury, or other pathological abnormalities (Figure 4).

## Discussion

Animal models form the cornerstone for investigating pathophysiological mechanisms in LTx.^23^ The orthotopic left lung transplantation model in rats remains widely adopted due to its technical simplicity; however, it does not fully reflect the physiological reality of single-lung transplantation in clinical settings. In rats, the left lung comprises only 1 of 5 lobes. Consequently, the compensatory function from the remaining 4 lung lobes allows survival despite alloimmune-mediated graft necrosis,^24^ a phenomenon distinct from human clinical scenarios. In contrast, the right lung contributes approximately 70% of total lung function in rats, making recipients highly dependent on the right graft following right lung transplantation. This dependency more closely mirrors human single-lung transplantation, where post-transplant survival is critically reliant on graft performance. However, the intricate anatomy of the rat right lung increases surgical complexity, prolongs donor lung ischemia times and compromises surgical success rates through associated complications.^11^, ^25^

Since Kawaguchi et al.’s seminal establishment of the rat orthotopic RLTx model in 1996,^6^ the progression and refinement of this model have demonstrated a relatively limited rate of advancement within the scientific community. A notable advancement by Li et al., introduced modified PV anastomosis through upper branch ligation and lower branch suturing, converting the conventional "4-branch" to a "3-branch" configuration, achieving a 7.5% success rate improvement.^19^ However, persistent challenges with the insufficiency in length of the venous cuff remained. Building upon our team's prior experience with the "pendulum model" in orthotopic left lung transplantation and the left-to-right inverted lung transplantation technique in rats, we developed a reverse-side model employing left-to-right inverted anastomosis to eliminate the compensatory effect from the native right lung.^13^, ^20^ Compared to conventional orthotopic left lung transplantation, this model eliminates the compensatory capacity of the native right lung, making recipient survival critically dependent on graft function and surgical precision rather than physiological reserve. However, the left-to-right inverted anastomosis presents inherent limitations, including non-physiological anatomical alignment and potential graft-thoracic size mismatch, highlighting the unique and irreplaceable advantages of standard right lung orthotopic transplantation.

Our optimized donor lung preparation overcame anatomical challenges by accounting for the confluence of the superior and inferior branches of the right pulmonary vein, which are located close to the left atrium and increases the variability for cuff insertion.^19^ We modified Li et al.’s approach by dissecting and ligating the left PV and transecting the right PV with partial left atrium resection, enabling PV branch convergence while preserving atrial tissue for cuff preparation. This approach resulted in only a minimal reduction in PV cuff length to 2 mm, compared to 3 mm in left lung transplantation. The donor preparation was 22.6 ± 1.8 minutes, and cold ischemia time was significantly reduced (38.3 ± 1.6 minutes vs 69.0 ± 8 minutes),^6^ with no bronchial/vascular ruptures or cuff displacements observed. With simplified surgical methods to maximize cuff length and controlled transverse incisions of the right PV, the ischemic time is significantly reduced, providing a solid foundation for the successful establishment of the model.

Vascular tearing during the anastomosis, another major challenge from spatial mismatches between vascular structures and cuffs,^26^ was mitigated through anterior-view PV/PA anastomosis followed by posterior-view bronchial anastomosis to minimize tension. Moreover, the complex anatomical structure of the right hilar is distinctive to the left hilum and significantly elevated the difficulty of the surgery. In this context, instead of using conventional nondestructive forceps, we employed multiple microvascular clips across vascular planes for proximal occlusion, reducing vascular tension and rupture risks despite slight stability trade-offs.

Right hilar vessels demonstrated thicker walls and larger luminal diameters compared to left counterparts, enabling controlled transverse incisions without tearing. In contrast to our previous model,^13^ this study employed transverse incision for all 3 hilar branches, with no instances of tearing observed. By employing the “pendulum model” to optimize the anastomotic sequence and vascular incisions, and integrating dual perspectives, among other key tricks, the shortest anastomosis time achieved was 18.3 minutes, with no vascular or bronchial injuries, reflecting a significant reduction in operation duration. Additionally, the mean anastomosis time was reduced to 20.2 minutes, markedly shorter than previously reported values.^18^, ^19^

Compared to other established RLTx models, the following technical refinements collectively contributed to improved surgical outcomes. As the right lung accounts for approximately 70% of the total pulmonary function, in this study, we used the survival rate of rats and, incidence of complications and histological assessment as representatives of graft function. Specifically, our model achieved a 90% survival rate, showed no significant abnormalities on histological examination, and demonstrated superiority over previously reported RLTx models.^18^, ^19^ This success is attributable to multiple factors: reduced donor lung ischemia time due to faster and more precise hilar dissection and cuff preparation, improved vascular alignment through dual-view anastomosis sequencing, and meticulous perioperative management, including pain control and close monitoring. Furthermore, the adoption of our previously validated “pendulum model” stabilization technique minimized tension and movement during anastomosis, enhancing graft stability and procedural reproducibility. These improvements, coupled with a notably elevated surgical success rate, establish a simplified and stable model for orthotopic RLTx.

This study acknowledges 2 limitations. Firstly, in the rat model, the accessory lobe of the right lung is physiologically located in the left thoracic cavity. Due to the focus on donor lung integrity, the transplanted lung’s placement in the recipient thorax does not guarantee optimal anatomical repositioning of the accessory lobe, potentially impacting the rat's prognosis. Secondly, although the surgical outcomes in this study were improved compared with previous models,^18^ these improvements cannot be attributed solely to the modifications we introduced. Rather, they likely reflect a combination of factors, including enhancements in the experimental environment, such as the use of advanced microsurgical tools, in addition to the modified techniques applied in our study.

## Conclusion

In this study, we refined the operational methodology for orthotopic RLTx, establishing a model characterized by reduced operation duration, minimized complications, and high success rates. This optimized approach offers a feasible and reproducible platform for rat LTx, serving as a valuable alternative for future research in the field.

## Financial support

This work was supported by the National Natural Science Foundation of China (General Program, Grant No. 82470104).

## Declaration of Competing Interest

The authors declare that they have no known competing financial interests or personal relationships that could have appeared to influence the work reported in this paper.

## Footnotes

Appendix A Supplementary data associated with this article can be found in the online version at doi:10.1016/j.jhlto.2025.100407.
