# Supplementary material for: A novel approach to establish orthotopic right lung transplantation in rats
Source: JHLT Open. 2025 Oct 10;10:100407. doi: 10.1016/j.jhlto.2025.100407 (PMC12605638; doi:10.1016/j.jhlto.2025.100407)
Supplement: Supplementary file 6 — Supplementary material [file mmc1.docx]

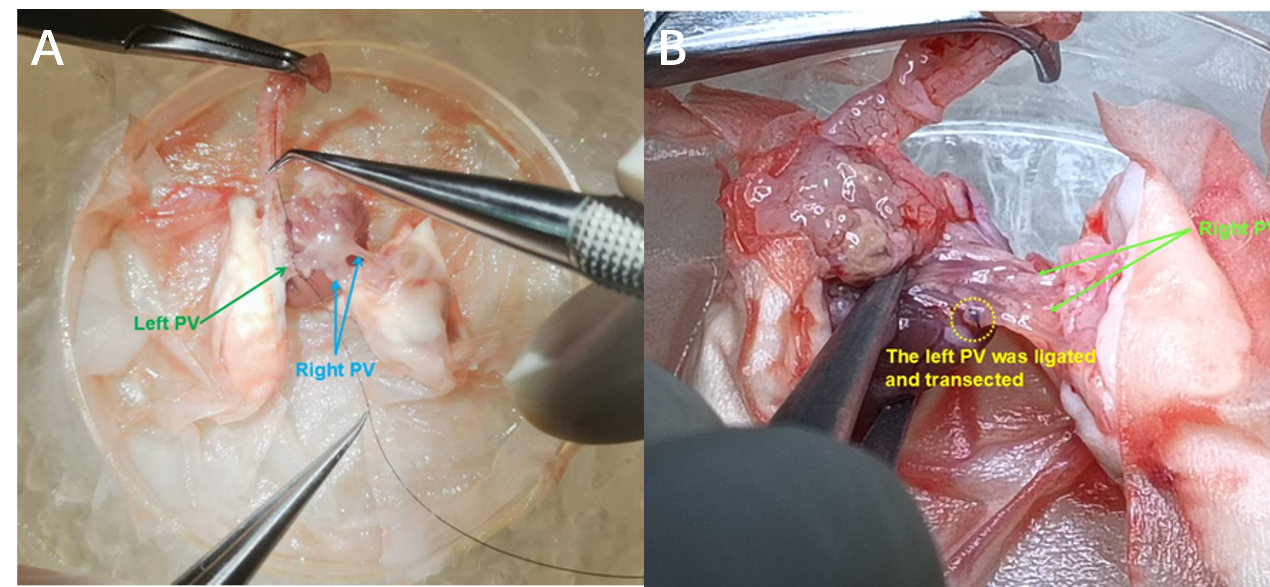
Supplementary figure 1. Anatomy of the pulmonary veins: (A) before and (B) after dissection of the left PV of donor lung.
